# Social behavior and spatial orientation in rat strains with genetic predisposition to catatonia (GC) and stereotypes (PM)

**DOI:** 10.18699/VJGB-22-35

**Published:** 2022-05

**Authors:** V.S. Plekanchuk, O.I. Prokudina, M.A. Ryazanova

**Affiliations:** Institute of Cytology and Genetics of the Siberian Branch of the Russian Academy of Sciences, Novosibirsk, Russia Novosibirsk State University, Novosibirsk, Russia; Institute of Cytology and Genetics of the Siberian Branch of the Russian Academy of Sciences, Novosibirsk, Russia; Institute of Cytology and Genetics of the Siberian Branch of the Russian Academy of Sciences, Novosibirsk, Russia

**Keywords:** catatonia, GC rat strain, PM rat strain, epilepsy, stereotypes, learning, social interaction, кататония, линия крыс ГК, линия крыс МД, эпилепсия, стереотипии, обучение, социальное взаимодействие

## Abstract

Various psychopathologies, including schizophrenia, bipolar disorder and major depression, are associated with abnormalities in social behavior and learning. One of the syndromes that may also take place in these disorders is catatonia. Catatonia is a psychomotor syndrome in which motor excitement, stereotypy, stuporous state, including the phenomenon of “waxy flexibility” (catalepsy), can be observed. Rats with genetic catatonia (GC) and pendulum-like movements (PM) of the anterior half of the body have physiological and behavioral changes similar to those observed in schizophrenia and depression in humans and can be considered as incomplete experimental models of these pathologies. The social behavior of the GC and PM rats has not been previously studied, and the cognitive abilities of animals of these strains are also insufficiently studied. To determine whether the GC and PM rats have changes in social behavior and spatial learning, behavioral phenotyping was performed in the resident-intruder test, three-chamber test, Barnes maze test. Some deviations in social behavior, such as increased offensive aggression in PM rats in the resident-intruder test, increased or decreased social interactions depending on the environment in different tests in GC, were shown. In addition, principal component analysis revealed a negative association between catatonic freezing and the socialization index in the three-chamber test. Decreased locomotor activity of GС rats can adversely affect the performance of tasks on spatial memory. It has been shown that PM rats do not use a spatial strategy in the Barnes maze, which may indicate impairment of learning and spatial memory.

## Abstract (ru)

Различные психопатологии, включая шизофрению, биполярное расстройство и большое депрессивное расстройство, ассоциированы с отклонениями в социальном поведении и обучении. Одним из синдромов, который также может иметь место при этих расстройствах, является кататония. Кататония – психомоторное расстройство, при котором могут наблюдаться двигательное возбуждение, стереотипии, ступор, в том числе с явлением «восковой гибкости» (каталепсии). Крысы с генетической кататонией (ГК) и маятникообразными стереотипными движениями (МД) имеют физиологические и поведенческие изменения, сходные с наблюдаемыми при шизофрении и депрессии у человека, и могут рассматриваться как неполные экспериментальные модели этих патологий. Социальное поведение крыс линий ГК и МД ранее не было исследовано, также недостаточно изучены когнитивные способности животных данных линий. Чтобы определить, имеются ли изменения в социальном поведении и пространственном обучении у крыс линий ГК и МД, было проведено их поведенческое фенотипирование в тесте «резидент-интрудер», трехкамерном тесте и лабиринте Барнс. В нашей работе показаны некоторые отклонения в социальном поведении, такие как усиление оборонительной агрессии у крыс МД, увеличение или уменьшение уровня социальных взаимодействий в зависимости от условий тестирования у крыс ГК. Кроме того, анализ главных компонент выявил отрицательную связь между кататоническим застыванием и индексом социальности в трехкамер- ном тесте. Снижение двигательной активности крыс ГК может негативно влиять на выполнение заданий по оценке пространственной памяти. Показано, что крысы МД не используют пространственную стратегию в лабиринте Барнс, что может указывать на нарушение обучения и пространственной памяти

## Introduction

In psychiatric classification, there is an acute issue of division and diagnosis of individual nosological units. A lot of evidence pointing to the generally continuous nature of psychopathological variation versus discrete has been accumulated (Krueger et al., 2018). In DSM-5 (Diagnostic and Statistical Manual of mental disorders), there are many “spectra” and groups of disorders (Autism Spectrum Disorder, Schizophrenia Spectrum and Other Psychotic Disorders, Bipolar and related disorders), the symptoms of which overlap very strongly. The comorbidity observed between major depression and schizophrenia (Samsom, Wong, 2015), bipolar disorder, attention deficit hyperactivity disorder, and autism (Kiser et al., 2015) implies that the same pathophysiological processes occur in these diseases. In this regard, new concepts are being created that try to explain the pathogenesis of distinct psychiatric symptoms and emphasize the exploration of endophenotypes but not of complex diseases (Anderzhanova et al., 2017). This approach solves the “problem of comorbidity” by explicitly modeling patterns of co-occurrence among signs and symptoms (Krueger et al., 2018).

One of the syndromes that can be used as a “specifier” in DSM-5 for the characterization of several clinical phenotypes including schizophrenia spectrum disorders, affective, and neurodevelopmental disorders is catatonia (Wilson et al., 2015). Catatonia is a psychomotor syndrome characterized by various signs: stupor, catalepsy (posturing, waxy flexibility), stereotypy, mutism. This motor and behavioral alteration may occur in many psychiatric conditions but predominantly in schizophrenia, affective psychosis, autism (Fink, Taylor, 2001). While many aspects of human psychopathologies cannot be simulated in animals, some symptoms of catatonia can. Different animal models can help characterize the nature of specific psychopathology symptoms, and there are special behavioral parameters of potential relevance to signs and symptoms of schizophrenia. Excessive catatonic reactions in animals can also correspond to catatonia in humans and include presence of bizarre motor activity, decrease in motor activity, or catatonic excitement (intense bursts of agitated stereotypy).

**Fig. 1.**
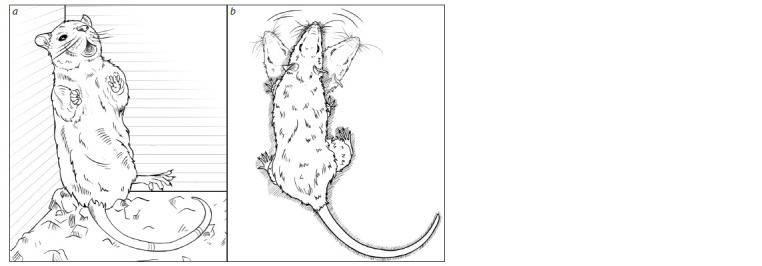
Catatonic reactions in rats: a, catalepsy in GC; b, pendulum-like movements of the PM rats.

For genetically based modeling of schizophrenia-relevant and catatonia-relevant symptoms, the GC (genetic catatonia) and the PM (pendulum-like movements) rat strains were offered (Timofeeva, 1985). The strains were obtained by selection for intensification of such catatonic reactions as freezing or catalepsy (GC strain) and stereotyped pendulum movements (PM strain). The GC rats demonstrate occasional freezing or, instead, hyperkinetic behavioral reactions that resemble the manifestations of the catatonic syndrome (Ryazanova et al., 2012). These reactions can be spontaneous, as well as in response to a weak stressful stimulus, such as in a special test for catatonic freezing (Fig. 1, a). In addition, rats of this strain are characterized by increased stress reactivity (Alekhina et al., 2015), increased shock-induced aggression (Nikulina et al., 1987), impaired filtration of sensorimotor information (manifested by a deficiency of PPI) (Ryazanova et al., 2017). PM rats are characterized by rhythmic side-to-side rocking of the head and forebody in the absence of locomotion (see Fig. 1, b). More than that, rats selected for an increased amplitude of pendulum-like movements after the 40th generation started generating seizures to audiogenic stimulation (Alekhina et al., 2007).

Despite some parameters supporting face validity of this model, phenotype of these strains is not yet well explored. For example, aspects of behavior and cognitive activity such as social interactions and learning are also of interest. A variety of neuropsychiatric disorders are characterized by disruptions in social behavior and social recognition, including depression, autism spectrum disorders, bipolar disorders, obsessive-compulsive disorders, and schizophrenia. In animals, altered social interaction responses in a variety of situations are considered as analogs related to negative – social withdrawal – symptoms of schizophrenia (Powell, Miyakawa, 2006), hyperactivity and aggressive behavior directly related to positive symptoms of schizophrenia (Volavka, Citrome, 2008).

To determine whether selection for predisposition to the catatonic freezing and the amplitude of pendulum-like movements influenced social interactions and learning in the GC and PM rat strains, behavioral phenotyping of rats in the resident intruder test, three-chamber test, Barnes maze test was carried out.

## Materials

The study was carried out on male rats of the GC (genetic catatonia), PM (pendulum-like movements), Wistar and WAG (Wistar Albino Glaxo) strains. Since the PM rat strain is outbred, rats of the outbred Wistar strain were used as a control, while for the inbred GC, the inbred WAG were used.

Experiment 1 included the catatonic freezing test (22 males at the age of 2 months from each strain); the three-chamber paradigm test (the same 15 males at the age of 5 months from each strain) and the resident-intruder test (5 days after the three-chamber test). In the Experiment 2, another 60 rats (15 males at the age of 4 months from each strain) were tested in the Barnes maze

Rats were kept under standard vivarium conditions with a free access to food and water. All experimental procedures complied with the rules and regulations formulated in the EU Council Directive 1986 (86/609/EEC) and the Declaration of Helsinki on the protection of vertebrate animals used in experimental research and approved by the ICG SB RAS Bioethics Committee (protocol No. 43, 28.09.2018).

Experiment 1. Social behavior and catatonia

The catatonic freezing test is a selection criterion for the GC rats and was carried out according to the protocol (Timofeeva, 1985). To determine the presence or absence of freezing reactions and their duration, the rat was uplifted in the corner of the cage by the forelegs using a test stick. The freezing time was estimated as a time during which the animal retained the induced posture or freezing position on 4 paws after the stick was removed. Rats were tested two times on different days.

The three-chamber paradigm test. The three-chamber social interaction assay was performed to assess social deficits according to the protocol (Kaidanovich-Beilin et al., 2011). Testing was carried out in a test arena manufactured by OpenScience, Russia, model TS1701-R. The apparatus for the test is comprised of a rectangular, three-chamber box. Each chamber is 40 × 85 cm and the dividing walls are made from clear Plexiglas, with an open middle section, which allows free access to each chamber. For habituation, the test rat was placed into a Plexiglas’s arena containing two empty cylindrical containers in two side chambers for 10 minutes.

Session I. Wistar males of the same weight without any prior contact (not littermates) with the subject were used as control animals (Stranger 1 and Stranger 2). One of the control rats (Stranger 1) was placed in one of the containers located in one of the side chambers. The placement of Stranger 1 on the left or right side of the chamber was systematically altered between trials. After removing the walls between the compartments, the following parameters were monitored and recorded: duration of direct contacts of the subject rat with Stranger 1; duration of contacts with empty enclosure. The duration of session I was 10 minutes. Then the Session II began. The second control rat was placed in the empty cylinder in the opposite side chamber. Duration of direct contacts of the subject rat with Stranger 1 and Stranger 2 were monitored and recorded within 10 minutes. The socialization index was calculated by the formula (T1 – T0)/(T1 + T0)×100 %, where T1 is the time of contact with the containment cup housing Stranger 1 rat; T0 – time of contact with the empty enclosure. The social novelty index is calculated by the formula (T2 – T1)/(T2 + T1)×100 %, where T1 is the time of contact with familiar rat (Stranger 1), T2 is the time of contact with the container housing Stranger 2 rat. The freezing time in this test was also recorded at each session (Freezing 1 and Freezing 2, respectively)

The resident-intruder test. To measure offensive aggression, the resident-intruder test was performed according to the standard protocol (Koolhaas et al., 2013). To assess the defensive behavior of resident males of the studied strain, they were placed in cages for 7 days before the test. To preserve olfactory signals, the cage was not cleaned before the test. The intruder (the Wistar male of the same size) was placed in the resident’s cage through the partition, then the partition was removed. Testing was carried out for 10 minutes. Durations of the behavioral parameters were registered: (1) total offense: sum of lateral threat, upright, clinch and keep down; (2) social exploration: sum of social explore, ano-genital sniffing and move towards; (3) non-social activity: non-social explore, rearing, grooming; (4) inactivity, including rest and freezing (freezing in the RI). Also the numbers of mounts and attack latencies were analyzed.

The analysis of the main factors determining the variability of behavior characteristics in Experiment 1 was investigated by the Principal Component Analysis.

Experiment 2. Spatial learning

The Barnes maze was used to test the acquisition of spatial memory. Testing was carried out in a setup manufactured by RPC OpenScience, Russia, model TS1101-R (field diameter 122 cm, 18 holes are located around the perimeter). Testing in the Barnes maze included 3-minute training sessions once a day for 5 days (Stansley, Yamamoto, 2015). Probe trial was administered 24 hours after the acquisition session (Day 7). The following parameters were to be calculated: (1) primary latency, (2) primary errors, (3) distance moved (in cm), and (4) velocity (cm/s) (Gawel et al., 2019).

Video tracking and registration of behavioral parameters were carried out using the program EthoVision XT 15 (Noldus, Wageningen, Netherlands). In addition, on 4, 5 and 7 day trial was classified into 1 of 3 categories of search strategy (Yassine et al., 2013) reflecting the use of either a direct spatial strategy (defined as direct visit to the target, sometimes preceded by at most 1 adjacent hole visit), a serial strategy (minimum of 2 adjacent hole visits in a serial manner before reaching the target) or a mixed (i. e., random) strategy (remaining trials). The data were subsequently analyzed in terms of percentage of trials with a direct spatial strategy

## Statistics

The obtained data were processed using STATISTICA 10.0. In the paper, data are presented as mean ± SEM. Behavioral scores from Experiment 1 were analyzed by Student t-tests (except for parameters: lateral threating, clinch attack, attack latencies, which was analyzed by Mann–Whitney U test). When comparing a rate, Fisher’s exact test was used. The analysis of the main factors that determine the variability of behavior characteristics in Experiment 1 was investigated by the principal component analysis. In Experiment 2, comparisons of components were made using the Mann–Whitney U test. Data analysis from the training sessions of Barnes maze was carried out using repeated measures ANOVA, followed by Fisher LSD post hoc analyses to analyze group differences. Statistical evaluation of the probe trial data was performed using one-way ANOVA, Fisher LSD post hoc analyses

## Results

Experiment 1. Social behavior and catatonia

The catatonic freezing test revealed a mean duration of freezing in the GC and PM rats is by far longer than in the control rats (35.6 ± 3.4 s in GC vs 18.4 ± 3.0 s in WAG, p < 0.001; 23.7 ± 3.7 s in PM vs 16.6 ± 4.5 s in Wistar, p < 0.05). In addition, a rate of rats in populations that freeze for longer than 10 seconds was estimated. In the GC (95.5 %) and PM (77.3 %) strains, the rate is significantly higher than in the control strains (63.6 and 39.1 %, respectively; р < 0.01, F = 0.0351 for GC; F = 0.0155 for PM).

A study of behavior in the three-chamber paradigm test showed a decrease in the sociability index in the GC rats (18.6 ± 10.2) compared to WAG (56.0 ± 10.1) (p < 0.05). The sociability index in the PM rats (35.7 ± 15.4 vs 44.6 ± 14.6), as well as the social novelty index in both groups (–9.4 ± 12.1 in GC, –0.7 ± 15.8 in PM) did not differ from the control (–15.8 ± 12.9 in WAG, –29.6 ± 12.5 in Wistar)

**Fig. 2.**
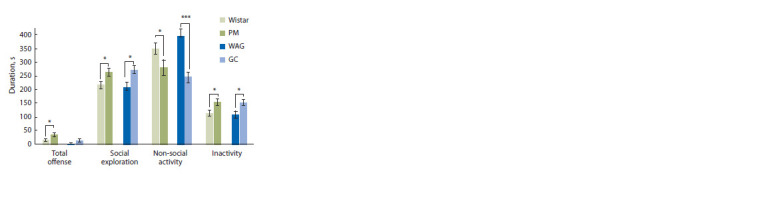
Behavioral profile of resident males during a ten minutes’ residentintruder test. Differences marked are shown for PM versus Wistar rats, and GC versus WAG. * p <0.05; *** p < 0.001, Student’s t-test.

In the resident-intruder test the parameters of resident’s behavior in the home cage when adding an intruder were registered and combined in categories (see Methods). The analysis revealed an increased level of social exploration of PM versus Wistar, as well as GC compared to WAG (p < 0.05) (Fig. 2). Moreover, unlike the GC, PM rats exhibited more aggressive behavior both in total duration (p < 0.05) and short attack latencies (90.3 ± 16.9) compared to Wistar (273.7 ± 65.8, p <0.05). In addition, the GC and PM strains showed significantly increased sexual behavior (p < 0.01), which was estimated in the number of mounts (3.5 ± 0.9 in GC vs 0.0 ± 0 in WAG; 2.9 ± 0.7 in PM vs 0.6 ± 0.4 in Wistar). Non-social activity of the GC and PM rats was significantly lower compared to control (p < 0.05 and p < 0.001), while the time of inactivity was higher (p <0.05). Thus, the behavior of the GC and PM rats in the home cage when the intruder is placed shifts towards an increase in social interactions with a decrease in exploratory activity.

The principal component analysis of Experiment 1 parameters produced three factors with eigenvalues greater than 1. These three factors explain 57 % of the variance in the correlation matrix. The factor patterns are presented in the Table.

**Table.**
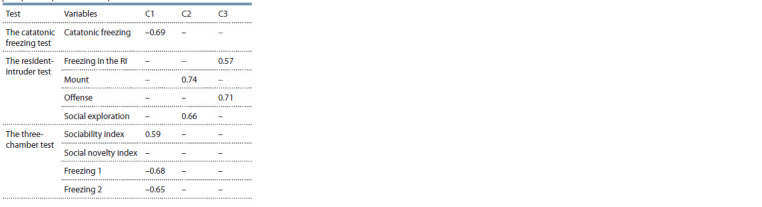
Component patterns for Experiment 1 Note. Catatonic freezing – duration of stupor in the catatonic freezing test; Freezing in RI – duration of immobility in the resident-intruder test; Mount – number of mounts in the resident-intruder test; Offense in RI – total duration of aggressive behavior in the resident-intruder test; Social exploration in RI – total duration of non-aggressive social behavior in the resident-intruder test; Sociability index – in the three-chamber paradigm test; Social novelty index – in the three-chamber paradigm test; Freezing 1 and Freezing 2 in the threechamber test – duration of immobility in the session I and in the session II, respectively, of the three-chamber paradigm test. Only component patterns above 0.55 were recorded

Component 1 (24.1 % of variance) was explained by stupor in the catatonic freezing test (–0.69) and in the three-chamber paradigm test (–0.68 for session I; –0.65 for session II) and sociability index value (0.59).

Component 2 (18.7 % of variance) was mainly loaded by number of mounts (0.74) and total duration of non-aggressive social behavior (0.66) in the resident-intruder test.

Component 3 (14.2 % of variance) was loaded by the total duration of aggressive behavior (0.71) and duration of immobility (0.57) in the resident-intruder test.

**Fig. 3.**
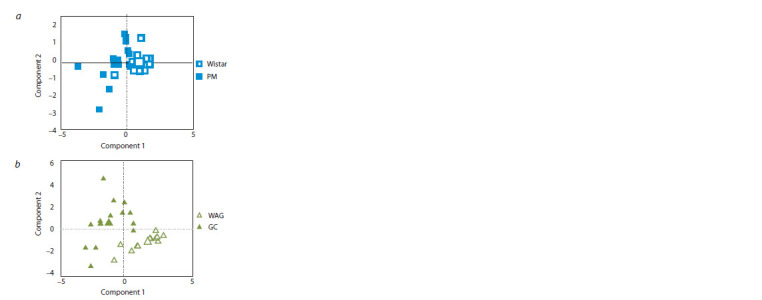
Principal component scores plot: a, the PM compared with Wistar. The mean scores of two principal components are indicated by larger squares; b, the GC compared with WAG. The mean scores of two principal components are indicated by larger triangles.

Mann–Whitney U test procedures showed a strain effect for Component 1 in PM and Wistar rats (p < 0.001) (Fig. 3, a). For WAG and GC, a significant difference was shown in Component 1 (p <0.001) and Component 2 (p < 0.01) (Fig. 3, b).

Experiment 2. Barnes maze task

**Fig. 4.**
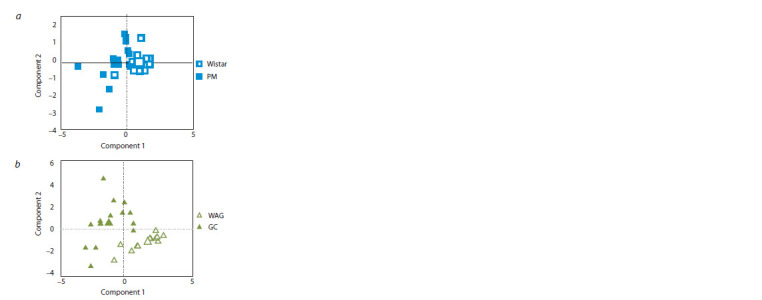
Spatial learning of the PM and GC rats during the acquisition session in the Barnes maze compared to Wistar and WAG, respectively: a, mean latencies to enter the escape hole; b, the average velocity of movement across the maze field; c, mean distance traveled; d, the incidence of spatial strategy in groups. * p < 0.05, ** p < 0.01, *** p <0.001.

**Fig. 5.**
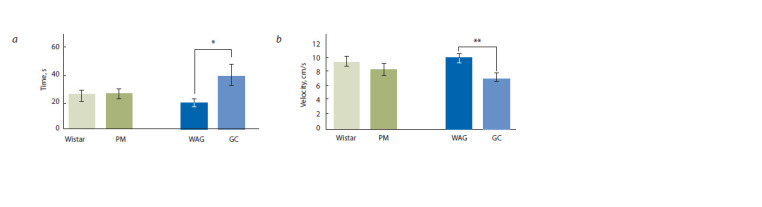
Probe trial in the Barnes maze of the PM and GC rats compared to Wistar and WAG, respectively: a, mean latencies to enter the escape hole; b, the average velocity of movement across the maze field. * p < 0.05, ** p < 0.01.

The data analysis revealed that latency time for the GC group was significantly increased in the probe trial when compared to the WAG group (F[1.26] = 5.9, p < 0.05) (Fig. 5, a). No difference was found between PM and Wistar. The average velocity of movement across the maze field did not differ for Wistar and PM. Comparison of GC and WAG rats speed revealed a significant effect of the test day (F[4, 108] = 13.7, p < 0.0001) and the interaction of factors of the genotype and the test day on the speed (F[4, 108] = 3.95, p < 0.001) was found. The average velocity of movement across the maze field was significantly lower for GC in day 3 (effect of the genotype factor, p < 0.001), in day 4 (p < 0.05), in day 5 (p < 0.05) (Fig. 4, b) and in the probe trial (F[1.28] = 12, p < 0.01) (see Fig. 5, b). Total distance moved did not differ between groups. The use of spatial strategy increased with the training during the acquisition phase, except for the PM group: in the 7th day of trials, the incidence of spatial strategy in the PM rats was 0 % (0/15) compared to 46.7 % (7/15) in the Wistar rats (p < 0.01, F = 0.0063, Fisher’s exact test) (see Fig. 4, d).

There was no significant effect of genetic group on the mean number of errors per trial made during the probe trial.

## Discussion

Experiment 1. Social behavior and catatonia

Decreased sociability in the three-chamber test shown by GC rats in this work is consistent with literature data about social abnormalities in different animal models of psychopathologies. Most of the animal models of schizophrenia have decreased or normal social interaction (Jones et al., 2011; Nani et al., 2019). In particular, in the model of negative symptoms of schizophrenia in animals induced by NMDA-receptor antagonists, social interaction deficits have been shown (Neill et al., 2010). DISC-1 mutations known to cause schizophrenialike abnormalities in rodents can impairs cognitive and social behaviors in some transgenic mice (Shevelkin et al., 2017; Sultana, Lee, 2020), but not in rats (Li, Zhang, 2017; Glenn et al., 2021). Research of knockouts of the Neuregulin-1 (NRG1) gene which has been identified as a candidate susceptibility gene for schizophrenia, revealed a selective impairment in response to social novelty in NRG1 mutants, but not in sociability (O’Tuathaigh et al., 2007). Developmental models of schizophrenia, such as using neonatal lesions of the rat ventral hippocampus or prenatal administration of methylazomethanol into pregnant rats, result in deficits in social behavior, as well as impaired memory, and increased anxiety (Sams-Dodd et al., 1997; Winship et al., 2019). Another selective breeding model of psychopathology that exhibits increased freezing to context (but unlike rats of the GC strain only onto acute prior stress) is the Wistar Kyoto (WKY) rats (Nosek et al., 2008). WKY is a depression model characterized by elevated anxiety- and depression-like behavior. In the social interaction assessment, the WKY rats avoided contact with another rats (Nam et al., 2014).

However, in the resident-intruder test, the total time that the GC and PM rats spent on direct contact with a social object (intruder) was significantly higher than that of control. This discrepancy in social activity in the two tests may be explained by different environmental conditions that affect emotional state and motivation. Stress level of the threechamber test is mostly caused by placing the experimental animal into a novel environment by the experimenter. Earlier it was shown that the GC rats react more strongly even to the handling required to place the animal in the experimental setup: corticosterone concentrations were increased during handling, but reduced at rest (Alekhina et al., 2016). Such an increased stress reactivity in the GC rats may explain the decrease in sociability index in the three-chamber test due to the passive-defensive reaction in response to handling stress in the three-chamber test, but not in the resident-intruder test, which does not require handling. Previously, it was shown that passive-defensive reflex expressed in the form of catatonic stupor is of dominant character and significantly prevails over cognitive and alimentary reflexes (Petrova, 1990). The results of this work suggest that the predisposition to catatonic stupor also negatively affects social motivation during testing in the three-chamber paradigm.

The data on the increase in social contacts of both GC rats and PM rats in the resident-intruder test shown in this work are of interest. It is known that an increase in social interaction in rodents can be achieved in certain ways, such as medial prefrontal cortex lesions (Gonzalez et al., 2000) or low doses of ethanol (Varlinskaya et al., 2001). At the neurochemical level, a wide variety of systems have been examined for their role in the normal expression of social behavior (Crowley et al., 1989). Oxytocin, vasopressin, endogenous opioids and catecholamines appear to participate in a wide variety of affiliative behaviors (Nelson, Panksepp, 1998). Acute administration of opiate drugs, low dose morphine and naltrexone produced a more robust attenuation of social investigation than non-social exploratory activity in rats. Amphetamine increased both forms of investigation and haloperidol had the opposite effect (Deak et al., 2009). More than that, there is evidence of the involvement of the glutamate system in the formation of social deviations. D-Cycloserine, a partial agonist at the glycine recognition site of the glutamatergic NMDA receptor, can increase social investigation and sexual behavior and decrease aggressiveness in mice (McAllister, 1994). There are results supporting a role of glutamate receptors subunits in the modulation of social behavior (Vekovischeva et al., 2004; Adamczyk et al., 2012), however the study of the glutamate receptors genes mRNA in the hippocampus and frontal cortex of the GC rats did not reveal any changes (Plekanchuk, Ryazanova, 2021).

The increased mounting in the GC and PM rats shown in this paper may be indicative of aggressiveness between rats of the same sex. It has previously been shown that the GC rats demonstrate a high level of shock-induced aggression (Nikulina et al., 1987), but not aggression towards male rats or interspecies aggression towards mice (Alekhina et al., 1987). In addition, both PM and GC rats have an increased aggressive response towards humans (Alekhina et al., 2016; Alekhina, Kozhemyakina, 2019).

Considering the fact that PM rats, in addition to catatonic symptoms, have a predisposition to audiogenic seizures, the connection between epilepsy and psychopathology in humans should be mentioned. Many symptoms of neurologic or psychiatric illnesses – such as cognitive impairment, depression, anxiety, attention deficits – occur more frequently in people with epilepsy than in the general population (Brooks‐Kayal et al., 2013). The rat lines selectively bred for differences in amygdala excitability, manifested by “fast” or “slow” kindling epileptogenesis, display several comorbid features related to anxiety and learning. Seizure-prone genetic background provides poorer original learning and easier disruption of new learning, as well as increased anxiety and impulsivity (McLntyre et al., 2004). Rats in the chronic phase of the lithium-pilocarpine model of epilepsy showed disturbed communicative behavior, with impaired social behavioral patterns, increased motor activity and impaired memory function (Smolensky et al., 2019). Aggression is one of several psychiatric disorders that is observed, among others, in epileptic patients (Deb et al., 2020). This association has been reliably replicated in several animal models including those using pilocarpine (Desjardins et al., 2001) and domoic acid (Fuquay et al., 2012), in which aggression develops either in parallel to spontaneous seizures or precedes the development of recurrent seizures. The increased offensive behavior of the PM rats in the resident-intruder test shown in this work may confirm the likely relationship between seizure predisposition and aggressiveness.

Experiment 2. The Barnes maze task

Rodent basal cognitive abilities include, along with elementary logic tasks solutions and generalization capacity of a low level, spatial behavior and memory. This type of cognitive ability requires the formation of mental representations of spatial environmental characteristics (Poletaeva, Zorina, 2014). To test the acquisition of spatial memory in PM and GC, the Barnes maze was used. The increased time required to search for the target hole in the GC rats may indicate impaired spatial learning. However, a decreased locomotor activity has earlier been shown in rats of this line (Petrova, 1990), and to assess whether potential disturbances are in fact memory impairments it is necessary to take into account such parameters as primary errors and search strategy. No differences were shown for these parameters in GC compared to WAG. The reduced GC rats velocity of movement across the maze field for 3–7 days confirms the effect of motor activity on latency to first target visits. The GC rats appear to have no learning impairment in this test. The fact that the velocity of movement of the GC rats in the field does not differ in the first two days of testing, but is less than in the control on the following days, may indicate a slower adaptation to new conditions.

Estimation of the search strategy showed differences in PM in comparison with Wistar. After a few days of training, non-cognitively impaired animals frequently use the spatial strategy to resolve the BM task. The fact that after a few days of learning trials the PM rats still use mixed (i. e., random) and serial strategies instead of spatial to resolve the maze means that they are cognitively impaired and do not employ spatial clues to reach the target hole (Yassine et al., 2013). It has previously been shown that the PM rats exhibit longer latency and lower rate of successful trials in the Morris water test, at the same time, the GC rats did not differ from the control in these parameters (Barykina et al., 2009). The Morris water maze is more stressful for animals than the Barnes maze, because there is water immersion (Gawel et al., 2019). Water-maze training induced greater increases in plasma corticosterone which may affect the performance of animals (Harrison et al., 2009). In addition, the GC rats are inclined to passive drift and longer floating episodes in the Morris water test (Barykina et al., 2009). Low movement speed in Barnes’s maze, high time of inactivity and low exploratory activity in the residentintruder test in the GC rats are caused by catatonic freezing. The data shown in this work confirm the manifestation of catatonic inhibition by the GC rats in different stressful situations (Barykina et al., 2009).

## Conclusion

Selection for the duration of catatonic freezing and the amplitude of pendulum-like movements influenced social interactions and learning in the GC and PM rat strains. In particular, the GC rats have increased or decreased social interactions depending on the environment, and a negative relationship between catatonic freezing and sociality were shown in this work. The PM rats show increased social activity and offensive aggression in the resident-intruder test. Except for the reduced velocity of movement across the maze field, the GC rats appear to have no difficulty in solving the Barnes maze, whereas the PM rats do not use a spatial strategy in the maze, which may indicate impairment of learning and spatial memory.

## Conflict of interest

The authors declare no conflict of interest.

## References

Adamczyk A., Mejias R., Takamiya K., Yocum J., Krasnova I.N.,
Caldero J., Wang T. GluA3-deficiency in mice is associated with
increased social and aggressive behavior and elevated dopamine
in striatum. Behav. Brain Res. 2012;229(1):265-272. DOI 10.1016/
j.bbr.2012.01.007

Alekhina T.A., Kozhemyakina R.V. Modeling of focal seizures with automatisms
in rats with pendulum movements. Bull. Exp. Biol. Med.
2019;168(2):300-303. DOI 10.1007/s10517-019-04695-7.

Alekhina T.A., Palchikova N.A., Igonina T.N., Kuznetsova N.V. Comparative
analysis of imipramine intake reactions in catatonic and wistar
rats. Rossiiskii Fiziologicheskii Zhurnal im. I.M. Sechenova =
Russian Journal of Physiology. 2015;101(3):249-257. (in Russian)

Alekhina T.A., Palchikova N.A., Kozhemyakina R.V., Prokudina O.I.
The signs of destabilization in behavioral and somatovegetative parameters
of rats selected for catatonia. Russ. J. Genet. Appl. Res.
2016;6(8):798-803. DOI 10.1134/S2079059716080025

Alekhina T.A., Prokudina O.I., Ryazanova M.A., Ukolova T.N.,
Barykina N.N., Kolpakov V.G. Typological characteristics of behavior
in strains of rats bred for enhancement and absence of pendulum
movements. Association with brain monoamines. Zhurnal Vysshey
Nervnoy Deyatel’nosti im. I.P. Pavlova = I.P. Pavlov Journal of
Higher Nervous Activity. 2007;57(3):336-343. (in Russian)

Alekhina T.A., Shtilman N.I., Nikulina E.M., Pavlov I.F., Barykina N.N.
Aggression and learning in a strain of rats predisposed to catalepsy.
Zhurnal Vysshey Nervnoy Deyatel’nosti im. I.P. Pavlova = I.P. Pavlov
Journal of Higher Nervous Activity. 1987;37(3):537-541. (in Russian)

Anderzhanova E., Kirmeier T., Wotjak C.T. Animal models in psychiatric
research: the RDoC system as a new framework for endophenotype-
oriented translational neuroscience. Neurobiol. Stress.
2017;7:47-56. DOI 10.1016/j.ynstr.2017.03.003.

Barykina N.N., Chugui V.F., Alekhina T.A., Ryazanova M.A., Ukolova
T.N., Sakharov D.G., Kolpakov V.G. Learning of rats predisposed
to catalepsy in Morris water test. Zhurnal Vysshey Nervnoy
Deyatel’nosti im. I.P. Pavlova = I.P. Pavlov Journal of Higher Nervous
Activity. 2009;59(6):728-735. (in Russian)

Brooks-Kayal A.R., Bath K.G., Berg A.T., Galanopoulou A.S., Holmes
G.L., Jensen F.E., Scharfman H.E. Issues related to symptomatic
and disease-modifying treatments affecting cognitive and
neuropsychiatric comorbidities of epilepsy. Epilepsia. 2013;54
(Suppl.4):44-60. DOI 10.1111/epi.12298

Crowley W.R., O’Connor L.H., Feder H.H. Neurotransmitter systems
and social behavior. In: Balthazart J. (Ed.) Molecular and Cellular
Basis of Social Behavior in Vertebrates. Advances in Comparative
and Environmental Physiology. Vol. 3. Berlin; Heidelberg; Springer,
1989;161-208. DOI 10.1007/978-3-642-73827-2_4.

Deak T., Arakawa H., Bekkedal M.Y., Panksepp J. Validation of
a novel social investigation task that may dissociate social motivation
from exploratory activity. Behav. Brain Res. 2009;199(2):326-333.
DOI 10.1016/j.bbr.2008.12.011.

Deb S., Brizard B.A., Limbu B. Association between epilepsy and
challenging behaviour in adults with intellectual disabilities: systematic
review and meta-analysis. BJPsych Open. 2020;6(5):e114.
DOI 10.1192/bjo.2020.96.

Desjardins D., Parker G., Cook L.L., Persinger M.A. Agonistic behavior
in groups of limbic epileptic male rats: pattern of brain damage
and moderating effects from normal rats. Brain Res. 2001;905(1-2):
26-33. DOI 10.1016/S0006-8993(01)02454-4.

Fink M., Taylor M.A. The many varieties of catatonia. Eur. Arch. Psychiatry
Clin. Neurosci. 2001;251(Suppl.1):I/8-I/13. DOI 10.1007/
pl00014200

Fuquay J.M., Muha N., Pennington P.L., Ramsdell J.S. Domoic acid
induced status epilepticus promotes aggressive behavior in rats.
Physiol. Behav. 2012;105(2):315-320. DOI 10.1016/j.physbeh.
2011.08.013

Gawel K., Gibula E., Marszalek-Grabska M., Filarowska J., Kotlinska
J.H. Assessment of spatial learning and memory in the Barnes
maze task in rodents – methodological consideration. Naunyn-
Schmiedeberg’s Arch. Pharmacol. 2019;392(1):1-18. DOI 10.1007/
s00210-018-1589-y.

Glenn M.J., Batallán Burrowes A.A., Yu W., Blackmer‐Raynolds L.,
Norchi A., Doak A.L. Progression of behavioral deficits during periadolescent
development differs in female and male DISC1 knockout
rats. Genes Brain Behav. 2021;e12741. DOI 10.1111/gbb.12741

Gonzalez L.E., Rujano M., Tucci S., Paredes D., Silva E., Alba G., Hernandez
L. Medial prefrontal transection enhances social interaction:
I: Behavioral studies. Brain Res. 2000;887(1):7-15. DOI 10.1016/
S0006-8993(00)02931-0.

Harrison F.E., Hosseini A.H., McDonald M.P. Endogenous anxiety
and stress responses in water maze and Barnes maze spatial
memory tasks. Behav. Brain Res. 2009;198(1):247-251. DOI 10.1016/
j.bbr.2008.10.015

Jones C.A., Watson D.J.G., Fone K.C.F. Animal models of schizophrenia.
Br. J. Pharmacol. 2011;164(4):1162-1194. DOI 10.1111/j.1476-
5381.2011.01386.x

Kaidanovich-Beilin O., Lipina T., Vukobradovic I., Roder J., Woodgett
J.R. Assessment of social interaction behaviors. J. Vis. Exp.
2011;48:e2473. DOI 10.3791/2473.

Kiser D.P., Rivero O., Lesch K.P. Annual research review: the (epi)genetics
of neurodevelopmental disorders in the era of whole‐genome
sequencing – unveiling the dark matter. J. Child Psychol. Psychiatry.
2015;56(3):278-295. DOI 10.1111/jcpp.12392.

Koolhaas J.M., Coppens C.M., de Boer S.F., Buwalda B., Meerlo P.,
Timmermans P.J. The resident-intruder paradigm: a standardized test for aggression, violence and social stress. J. Vis. Exp.
2013;77:e4367. DOI 10.3791/4367.

Krueger R.F., Kotov R., Watson D., Forbes M.K., Eaton N.R., Ruggero
C.J., Zimmermann J. Progress in achieving quantitative classification
of psychopathology. World Psychiatry. 2018;17(3):282-293.
DOI 10.1002/wps.20566.

Li M., Zhang M. SU10. Behavioral characteristics of a DISC1 knockout
rat model. Schizophr. Bull. 2017;43(Suppl.1):S164. DOI 10.1093/
schbul/sbx024.009

McAllister K.H. D-cycloserine enhances social behaviour in individually-
housed mice in the resident-intruder test. Psychopharmacology.
1994;116(3):317-325. DOI 10.1016/0031-9384(86)90007-7.

McLntyre D.C., McLeod W.S., Anisman H. Working and reference
memory in seizure-prone and seizure-resistant rats: impact
of amygdala kindling. Behav. Neurosci. 2004;118(2):314-323.
DOI 10.1037/0735-7044.118.2.314.

Nam H., Clinton S.M., Jackson N.L., Kerman I.A. Learned helplessness
and social avoidance in the Wistar-Kyoto rat. Front. Behav.
Neurosci. 2014;8:109. DOI 10.3389/fnbeh.2014.00109.

Nani J.V., Rodríguez B., Cruz F.C., Hayashi M.A.F. Animal models
in psychiatric disorder studies. In: Tvrdá E., Yenisetti S.C. (Eds.)
Animal Models in Medicine and Biology. IntechOpen, 2019.
DOI 10.5772/intechopen.89034.

Neill J.C., Barnes S., Cook S., Grayson B., Idris N.F., McLean S.L.,
Harte M.K. Animal models of cognitive dysfunction and negative
symptoms of schizophrenia: focus on NMDA receptor antagonism.
Pharmacol. Ther. 2010;128(3):419-432. DOI 10.1016/
j.pharmthera.2010.07.004.

Nelson E.E., Panksepp J. Brain substrates of infant–mother attachment:
contributions of opioids, oxytocin, and norepinephrine. Neurosci.
Biobehav. Rev. 1998;22(3):437-452. DOI 10.1016/S0149-
7634(97)00052-3.

Nikulina E.M., Popova N.K., Kolpakov V.G., Alekhina T.A. Brain dopaminergic
system in rats with a genetic predisposition to catalepsy.
Biog. Amines. 1987;4(4-6):399-406.

Nosek K., Dennis K., Andrus B.M., Ahmadiyeh N., Baum A.E.,
Woods L.C.S., Redei E.E. Context and strain-dependent behavioral
response to stress. Behav. Brain Funct. 2008;4(1):1-8.
DOI 10.1186/1744-9081-4-23.

O’Tuathaigh C.M.P., Babovic D., O’Sullivan G.J., Clifford J.J.,
Tighe O., Croke D.T., Waddington J.L. Phenotypic characterization
of spatial cognition and social behavior in mice with ‘knockout’of the
schizophrenia risk gene neuregulin 1. Neuroscience. 2007;147(1):
18-27. DOI 10.1016/j.neuroscience.2007.03.051.

Petrova E.V. Features of changes in congenital and acquired forms of
behavior in rats with genetic catalepsy. Zhurnal Vysshey Nervnoy
Deyatel’nosti im. I.P. Pavlova = I.P. Pavlov Journal of Higher Nervous
Activity. 1990;40(3):475-480. (in Russian)

Plekanchuk V.S., Ryazanova M.A. Expression of glutamate receptor
genes in the hippocampus and frontal cortex in GC rat strain with
genetic catatonia. J. Evol. Biochem. Physiol. 2021;57(1):156-163.
DOI 10.1134/s0022093021010154

Poletaeva I.I., Zorina Z.A. A genetic approach to the study of simple
cognitive abilities in animals. Rossiyskiy Zhurnal Kognitivnoy
Nauki = Russian Journal of Cognitive Science. 2014;1(3):31-55.

Powell C.M., Miyakawa T. Schizophrenia-relevant behavioral testing
in rodent models: a uniquely human disorder? Biol. Psychiatry.
2006;59(12):1198-1207. DOI 10.1016/j.biopsych.2006.05.008.

Ryazanova M.A., Igonina T.N., Alekhina T.A., Prokudina O.I. The increase
in the proportion of nervous animals bred for catatonia: the participation
of central adrenoreceptors in catatonic
reactions. Russ. J.
Genet. 2012;48:1141-1147. DOI 10.1134/S1022795412100092.

Ryazanova M.A., Prokudina O.I., Plekanchuk V.S., Alekhina T.A.
Expression of catecholaminergic genes in the midbrain and prepulse
inhibition in rats with a genetic catatonia. Vavilovskii Zhurnal
Genetiki i Selektsii = Vavilov Journal of Genetics and Breeding.
2017;21(7):798-803. DOI 10.18699/VJ17.296. (in Russian)

Sams-Dodd F., Lipska B.K., Weinberger D.R. Neonatal lesions of the
rat ventral hippocampus result in hyperlocomotion and deficits in
social behaviour in adulthood. Psychopharmacology. 1997;132(3):
303-310. DOI 10.1007/s002130050349.

Samsom J.N., Wong A.H.C. Schizophrenia and depression co-morbidity:
what we have learned from animal models. Front. Psychiatry.
2015;6:13. DOI 10.3389/fpsyt.2015.00013.

Shevelkin A.V., Terrillion C.E., Abazyan B.N., Kajstura T.J., Jouroukhin
Y.A., Rudow G.L., Pletnikov M.V. Expression of mutant
DISC1 in Purkinje cells increases their spontaneous activity and
impairs cognitive and social behaviors in mice. Neurobiol. Dis.
2017;103:144-153. DOI 10.1016/j.nbd.2017.04.008.

Smolensky I.V., Zubareva O.E., Kalemenev S.V., Lavrentyeva V.V.,
Dyomina A.V., Karepanov A.A., Zaitsev A.V. Impairments in cognitive
functions and emotional and social behaviors in a rat lithiumpilocarpine
model of temporal lobe epilepsy. Behav. Brain Res.
2019;372:112044. DOI 10.1016/j.bbr.2019.112044.

Stansley B.J., Yamamoto B.K. Behavioral impairments and serotonin
reductions in rats after chronic L-dopa. Psychopharmacology.
2015;232(17):3203-3213. DOI 10.1007/s00213-015-3980-4.

Sultana R., Lee C.C. Expression of behavioral phenotypes in genetic
and environmental mouse models of schizophrenia. Front. Behav.
Neurosci. 2020;14:29. DOI 10.3389/fnbeh.2020.00029.

Timofeeva A.S. (Ed.) Genetic and Evolutionary Problems in Psychiatry.
Novosibirsk: Nauka Publ., 1985. (in Russian)

Varlinskaya E.I., Spear L.P., Spear N.E. Acute effects of ethanol on
behavior of adolescent rats: role of social context. Alcohol. Clin.
Exp. Res. 2001;25(3):377-385. DOI 10.1111/j.1530-0277.2001.
tb02224.x.

Vekovischeva O.Y., Aitta‐aho T., Echenko O., Kankaanpää A.,
Seppälä T., Honkanen A., Korpi E.R. Reduced aggression in
AMPA-type glutamate receptor GluR-A subunit-deficient mice.
Genes Brain Behav. 2004;3(5):253-265. DOI 10.1111/j.1601-
1848.2004.00075.x.

Volavka J., Citrome L. Heterogeneity of violence in schizophrenia
and implications for long‐term treatment. Int. J. Clin. Pract.
2008;62(8):1237-1245. DOI 10.1111/j.1742-1241.2008.01797.x.

Wilson J.E., Niu K., Nicolson S.E., Levine S.Z., Heckers S. The diagnostic
criteria and structure of catatonia. Schizophr. Res. 2015;164(1-3):
256-262. DOI 10.1016/j.schres.2014.12.036.

Winship I.R., Dursun S.M., Baker G.B., Balista P.A., Kandratavicius
L., Maia-de-Oliveira J.P., Howland J.G. An overview of animal
models related to schizophrenia. Can. J. Psychiatry. 2019;64(1):
5-17. DOI 10.1177/0706743718773728.

Yassine N., Lazaris A., Dorner-Ciossek C., Després O., Meyer L., Maitre
M., Mathis C. Detecting spatial memory deficits beyond blindness
in tg2576 Alzheimer mice. Neurobiol. Aging. 2013;34(3):716-
730. DOI 10.1016/j.neurobiolaging.2012.06.016.

